# First person – Arístides López-Márquez

**DOI:** 10.1242/dmm.052771

**Published:** 2026-01-22

## Abstract

First Person is a series of interviews with the first authors of a selection of papers published in Disease Models & Mechanisms, helping researchers promote themselves alongside their papers. Arístides López-Márquez is first author on ‘ *Col6a1* knock-in mice provide a promising pre-clinical model for collagen-VI-related dystrophies’, published in DMM. Arístides is a senior postdoctoral researcher in the lab of Cecilia Jiménez-Mallebrera at Sant Joan de Deu Research Institute (Barcelona, Spain) investigating advanced personalized therapies based on nucleic acids, such as gene editing or antisense oligonucleotides, for congenital muscular dystrophies and rare genetic diseases.


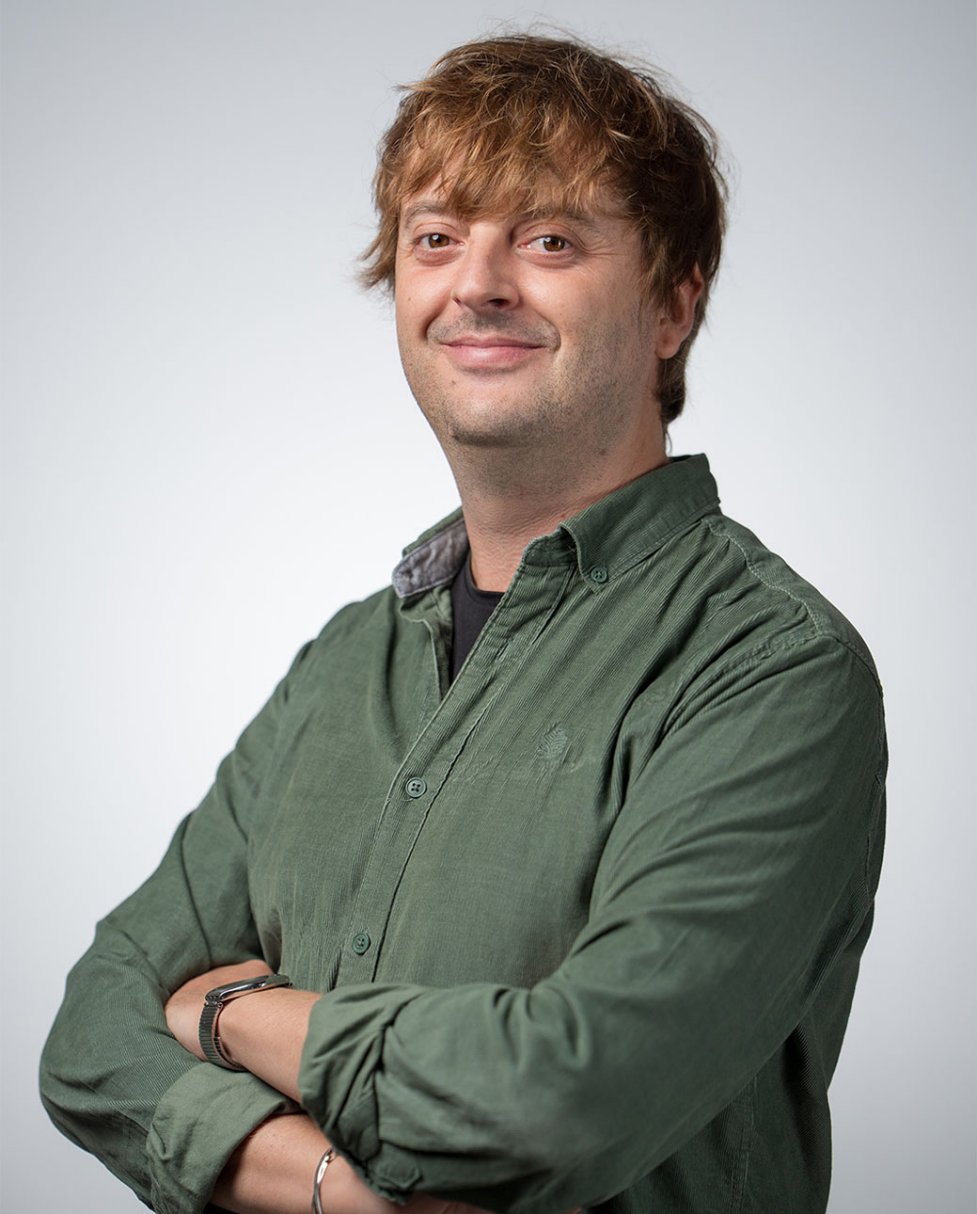


**Arístides López-Márquez**

**Who or what inspired you to become a scientist?**

From childhood, I was interested in understanding how the world around us worked: animals, plants, ecosystems. I remember my family always had animals, like cats, dogs, chickens and others, and I would carefully observe them to try to understand their interactions and their patterns of behaviour. But it was really my High School biology teacher, Matilde, who made a lasting impression on me by beautifully teaching me the magic of cell biology, genetics and biochemistry, and helping me understand how, by deciphering these processes, I could understand, explain and answer many of the questions I had always asked myself. That's when I knew I wanted to be a scientific researcher.

We have generated and characterized a mouse model carrying a *Col6a1* missense mutation that is equivalent to the variant observed in a large number of our patient cohort

**What is the main question or challenge in disease biology you are addressing in this paper? How did you go about investigating your question or challenge?**

In this paper we focus our work on a very rare congenital muscular dystrophy, collagen-VI-related disorder (COL6-RD), a pathology that seriously affects the quality and life expectancy of the patients who have it − usually children − and for which there is currently no effective treatment. COL6-RD is due to variations in the genes that encode collagen VI, i.e. *COL6A1*, *COL6A2* and *COL6A3*. Most of these variations are *de novo* and missense, with a dominant-negative effect on the correct assembly of collagen VI in the extracellular matrix, affecting skeletal muscle function. In this work, we have generated and characterized a mouse model carrying a *Col6a1* missense mutation that is equivalent to the variant observed in a large number of our patient cohort.

**How would you explain the main findings of your paper to non-scientific family and friends?**

Our research group has spent many years investigating potentially therapeutic strategies based on nucleic acids, such as gene editing to correct or silence pathogenic variants or antisense oligonucleotides. We have made significant progress using patient-derived fibroblasts; these are our target cells because they are responsible for the production of collagen VI. However, to further advance the development of these therapies, we need a disease model that recreates the pathogenic phenotype of this mutation, in which we can test and optimize these therapies (efficacy, safety, dosage, etc.). Furthermore, this mouse model will also be very useful for furthering our understanding of aspects of the disease's pathophysiology that are still not well understood − such as muscle fibrosis and the severe respiratory failure due to diaphragmatic dysfunction that patients experience − and that the model also reproduces.

**What are the potential implications of these results for disease biology and the possible impact on patients?**

Thanks to the results obtained in our work, after deep phenotypic and functional characterization of the mouse model generated and reported, we were able to learn in more detail aspects of the pathophysiology of this devastating muscular dystrophy, such as increased fibrosis in skeletal muscle or the impairment of respiratory function, one of the most serious limitations in COL6-RD patients. In addition to all this, and what is most promising, we anticipate this mouse model to be very useful for testing potentially therapeutic strategies not only based on silencing this specific mutation, but also other possible therapies that address other symptoms mimicked by our mouse model. Therefore, we sincerely hope this model will significantly help us advance towards the clinical application of the therapies we are investigating in our laboratory.

Our research group is very proud to have published in DMM, and we are convinced that DMM is an excellent platform to disseminate this murine COL6-RD model

**Figure:**
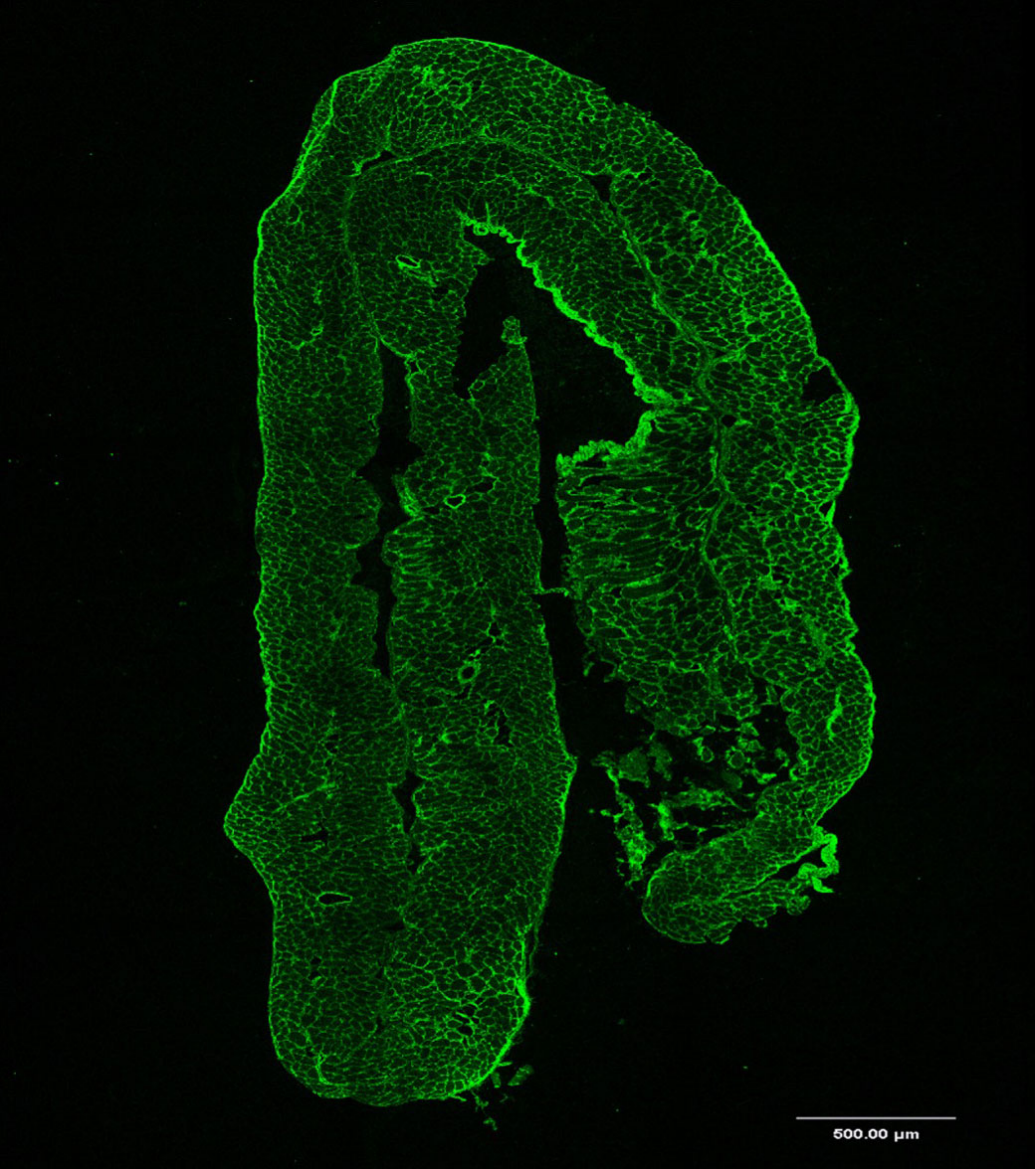
Immunostained cross section of the diaphragm from a mouse heterozygous for the pathogenic missense variant *Col6a1* c.874G>A, showing the expression pattern of collagen VI in the extracellular matrix.

**Why did you choose DMM for your paper?**

After these years of work, we were determined to report our animal model − the first for a dominant-negative missense mutation in COL6-RD − in a prestigious, high-impact journal for the scientific community. It goes without saying that DMM is a journal with a long history, and that it is one all researchers working in the field of disease models and pathological mechanisms consult regularly and with confidence. Our research group is very proud to have published in DMM, and we are convinced that DMM is an excellent platform to disseminate this murine COL6-RD model generated and characterized by us.

**Given your current role, what challenges do you face and what changes could improve the professional lives of other scientists in this role?**

On a scientific level, my main challenge is to continue researching to close the gap that still exists between the discoveries we make in the lab and their application to our patients − who are our primary reason for doing what we do. On a personal level, little by little and with the support of our group's principal investigator Cecilia Jimenez-Mallebrera, my main challenge is to gradually assume more responsibility, independence and leadership skills, so that I can establish my own line of research in the field of neuromuscular diseases and new advanced therapies. Regarding areas for improvement, greater access to funding and stable, long-term positions would significantly help young researchers, who are making their way in the world of research.

My main challenge is to improve the lives of my patients and their families, and I try to work towards that every day

**What's next for you?**

From now on, having generated and characterized the COL6-RD mouse model reported in DMM, a wide range of possibilities opens up for further optimization of the delivery systems for the nucleic acid-based therapies we are using. Therefore, we are focused on studying different types of vehicle in which to incorporate these tools − such as CRISR and ASOs − and to deliver them efficiently, safely and reproducibly to different skeletal muscles. In this regard, our major challenge is reaching very diverse muscles with many unique characteristics, such as the diaphragm or the quadriceps, given the systemic nature of COL6-RD. Meanwhile, we also have the ambition and conviction to continue understanding the molecular, cellular and physiological mechanisms involved in COL6-RD, as well as in other − equally severe − congenital muscular dystrophies. However, my main challenge is to improve the lives of my patients and their families, and I try to work towards that every day.

**Tell us something interesting about yourself that wouldn't be on your CV**

Outside of the lab, I try to have hobbies that help me broaden my mind and also, to some extent, relax. I especially enjoy reading, from novels to essays, cooking and music. Particularly classical music and flamenco, which is typical from Andalusia, the region in the south of Spain where I was born. In that sense, although I live in Barcelona, it's essential for me to be able to travel to my birthplace, Sanlúcar de Barrameda, an old and beautiful city next to the sea, where I can spend time with my family and friends, and enjoy its cuisine and beaches.
